# Crack Detection Zones: Computation and Validation

**DOI:** 10.3390/s20092568

**Published:** 2020-04-30

**Authors:** Simon Pfingstl, Martin Steiner, Olaf Tusch, Markus Zimmermann

**Affiliations:** 1 Laboratory for Product Development and Lightweight Design, Technical University of Munich, Boltzmannstr. 15, 85748 Garching, Germany; martintsteiner@web.de (M.S.); zimmermann@tum.de (M.Z.); 2 iABG, Einsteinstr. 20, 85521 Ottobrunn, Germany; tusch@iabg.de

**Keywords:** structural health monitoring, predictive maintenance, crack detection, fatigue damage, aerospace structures

## Abstract

During the development of aerospace structures, typically many fatigue tests are conducted. During these tests, much effort is put into inspections in order to detect the onset of failure before complete failure. Strain sensor data may be used to reduce inspection effort. For this, a sufficient number of sensors need to be positioned appropriately to collect the relevant data. In order to minimize cost and effort associated with sensor positioning, the method proposed here aims at minimizing the number of necessary strain sensors while positioning them such that fatigue-induced damage can still be detected before complete failure. A suitable detection criterion is established as the relative change of strain amplitudes under cyclic loading. Then, the space of all possible crack lengths is explored. The regions where the detection criterion is satisfied before complete failure occurs are assembled into so-called detection zones. One sensor in this zone is sufficient to detect criticality. The applicability of the approach is demonstrated on a representative airplane structure that resembles a lower wing section. The method shows that four fatigue critical spots can be monitored using only one strain sensor in a non-intuitive position. Furthermore, we discuss two different strain measures for crack detection. The results of this paper can be used for reliable structural health monitoring using a minimum number of sensors.

## 1. Introduction

Since fatigue-induced damage is still one of the most uncertain and unpredictable failure mechanisms [1], the development process of new structures requires many fatigue tests. The developer must assure safety over the entire lifetime. This is especially true for the aerospace industry, where the risk is high. In order to do so, certification tests are required. During the fatigue tests, the developer inspects the structure periodically to assure that no failures occur during runtime. This causes much effort, particularly for big structures e.g., a full-scale fatigue test, where an entire plane is tested on the ground.

Structural health monitoring (SHM) is the process of implementing a damage identification strategy for aerospace, civil, and mechanical engineering infrastructure [2]. Currently, data-driven approaches come more and more into practice for this application. Often the focus is on presenting new algorithms for predictive maintenance [3]. However, these approaches only work well if the collected data indicate the structural failure. This means that it is crucial to have a reliable dataset, which indicates the damage. Therefore, sensor positioning plays an important role in SHM [4].

The preferred sensor technologies are amongst piezo sensors, fiber Bragg gratings (FBG), and strain gauges (SG) [5]. For example, the acoustic emission (AE) technique allows the detection of a propagating crack by detecting and analyzing the high-frequency elastic waves emitted by the fracture process itself [6]. In [7,8], the authors use AE to monitor crack growth in compact tension specimens by counting the emissions occurring near the peak load. Different measures can be evaluated from the measured signal. However, a standardization of this approach is still progressing and certain thresholds are limited to specific specimens [9]. The information collected by an AE system allows one to determine both the location of the activity and the source of damage. In comparison with a strain-based approach, an advantage of AE is that larger areas can be monitored. However, the high ratio of acoustic signal damping in complex structures, as well as noise not being generated from the damage, can have a negative impact on the sensor signals. Therefore, it is difficult to practically interpret acoustic signals [10]. In [11], the authors apply different techniques, such as FBG and AE, to monitor cracks in masonry. It is stated that both FBG-type strain sensors and AE sensors give the most reliable and useful data sets for crack monitoring.

Recently, many researchers have been using FBGs for crack detection [12,13,14,15] and SHM [16,17] because of their good linearity and resistance to harsh environments [18]. Even their fatigue performance is superior to the alternative electrical resistance SGs [19]. It must be noted that this is related to the sensor itself and not to a sensor that is glued to a structure. Furthermore, compared to SGs, an advantage of FBGs is that they can be integrated into a composite structure and cover a large area of the structure [20]. However, FBGs have no standards for the sensor packaging and their usage in SHM [18]. Another disadvantage is the high cost of the interrogation system [18]. Since the sensor packaging and the application of the sensor are not the scopes of this paper, we use strain gauges for collecting the data. Moreover, many approaches for vibration-based SHM [21,22,23] including mathematical criteria for sensor positioning [24], as well as FBG approaches, are already explored, whereas, for SGs, the authors could find only a few publications. For example, in [25,26,27], SHM is applied with SGs, but either the sensor positioning method is not mentioned or the authors state that the sensors should be placed close to the expected crack initiation spot. This leads in [26] to many applied sensors. In contrast, for guided wave-based sensing [28] and accelerometer techniques [29], there are already sensor minimization approaches available. Therefore, this paper presents a method which

- proposes reliable sensor positions for SGs,
- tries to omit redundant sensors, and thus
- enables a passive SHM system.

It must be noted that the proposed method is not limited to SGs. The proposed method identifies those regions, where a strain sensor is necessary. This can be an SG, a part of an FBG, or a digital image correlation technique, which is applied e.g., in [30].

## 2. Proposed Method

For developing an SHM system, the failure mechanisms, its criteria, the possible failure locations, and the sensor system should be known. The general idea is to determine the change of the system response due to all possible failures. Then, the detection zones are identified by computing those regions, which indicate for every failure a certain change of the system response. In this paper, the authors consider thin-walled structures, where fatigue cracks can occur. The stress intensity factor (SIF) serves as the fracture criterion. A fatigue analysis, which is explained in Section 2.1., identifies the possible failure locations. Herein, strain sensors shall detect fatigue cracks. Hence, the change of strain serves as the system response indicating the failure. The proposed method employs the following assumptions:

- The method is developed and applied to a structure that is subjected to a plane stress condition.
- The crack direction is perpendicular to the maximum principal stress direction and a crack initiates at the position of maximum in-plane stress.
- The crack direction over the entire path is constant.

**Figure 1:**
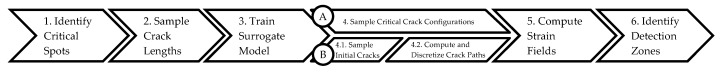
Process of the proposed method.

The general idea of the method is first to identify the fatigue critical locations. At these locations, possible sizes of failures are introduced in a computational model to compute the criticality of the failures. After finding critical failure modes by a surrogate model, the computational model is used to quantify the change of the system response. Finally, the detection zones are identified by overlapping for all critical failure modes those zones, which have a prescribed change of the system response. The detailed process of the method, depicted in Figure 1, has six building blocks, where the fourth is split into two paths, A and B. The following subchapter explains the basic process regarding the upper path. For path A, it is assumed that the cracks shall be detected when they get critical with respect to an SIF criterion. The lower path is an extension. It takes into account that cracks can be also detected on the way to its critical state. This is explained in Section 2.2.

### 2.1. Process for Crack Detection Zones

*Identify critical spots.* The proposed method begins with identifying critical spots of a structure with its geometry, maximum load, and boundary conditions, which points out where the structure is prone to fatigue failure. This can be done by using a finite element analysis (FEA), rainflow counting [31], Miner’s rule [32], and the corresponding S-N-curve.

*Sample crack lengths.* Beginning with multiple critical spots, secondly, different combinations of crack lengths are Monte Carlo sampled and put into the finite element model. It is important to regard also combinations that have zero crack lengths at a possible fatigue location. The authors use the extended finite element method (XFEM) and the virtual crack closure technique (VCCT) in the commercial software Abaqus (Johnston, SC, USA) to run the FEAs and evaluate the SIF at each crack tip. This step maps different combinations of crack lengths to the corresponding SIFs.

*Train surrogate model.* Thirdly, a surrogate model is trained to evaluate SIFs for crack length combinations quickly. An array of crack length combinations a serves as the input. The output is an array of SIFs K. Each component of this array belongs to the corresponding critical spot.

*Sample critical crack configurations.* Now, in step 4, the critical crack front (CCF), i.e., where at least one of the cracks becomes critical, can be computed. In this, a crack is regarded as critical when the SIF reaches the fracture toughness $K_{c}$. In order to detect the cracks before they get critical, a safety factor S is applied. The corresponding hyperplane is defined as the detectable crack front (DCF) and belongs to the criterion of Equation (1). For getting critical crack length configurations $a_{c}$, different combinations can be sampled and evaluated. If the criterion of Equation (1) holds by a certain accuracy, a point on the DCF is found:

(1) $$\max\left(K\right)=\frac{K_{c}}{S}$$

*Compute strain fields.* After computing a certain number of points on the DCF, the corresponding strain fields are evaluated by an FEA. From there, the absolute and the relative change of strain fields $\Delta \epsilon_{abs}$ and $\Delta \epsilon_{rel}$ can be computed with Equations (2) and (3), respectively:

(2) $$\Delta \epsilon_{abs}=\epsilon -\epsilon_{0}$$

(3) $$\Delta \epsilon_{rel}=\frac{\epsilon -\epsilon_{0}}{\epsilon_{0}}$$

*Identify detection zones.* By doing so for all points on the DCF and applying certain detection conditions for the minimum relative and absolute change of strain $\Delta \epsilon_{rel}$ and $\Delta \epsilon_{abs}$, detection zones for the corresponding crack combination can be found. By overlapping these zones, a final detection map can be determined.

### 2.2. Considering Crack Paths

Since a crack can also be detected on its way to a critical state, an extension to the beforehand process is introduced. Figure 1 depicts this in the lower path of building block four.

*Sample initial cracks.* The time of crack initiation is assumed to lie on the S-N-curve and follows a prescribed distribution, e.g., a log10-distribution. Scatter values for different materials can be found in [33] (p. 527). Now, the time of crack initiation is sampled from the corresponding distribution.

*Compute and discretize crack paths.* Since a surrogate model, which can evaluate that the SIFs for combinations of crack lengths are already established, the crack paths can be computed with the Paris law [34] of Equation (4), where C and $m^{*}$ are material parameters and ΔK is the range of the SIF. The Paris law represents also the gradient in the crack length space. After that, the crack paths are discretized into several points in the crack length space:

(4) $$\frac{da}{dN}=C\;\Delta K^{m^{*}}$$

For these points, the change of strain fields is evaluated by an FEA. By applying prescribed thresholds to the relative and absolute change of strain fields, the detection zones can be computed. Now, for every crack path, the unions of the detection zones are computed. After that, the intersection between all different crack paths represents the final detection map.

## 3. Validation of Critical Crack Front

**Figure 2:**
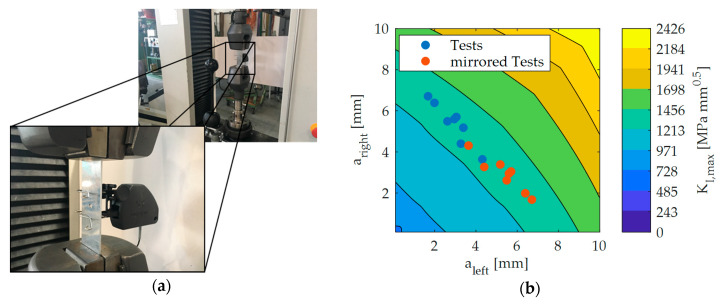
(**a**) test setup; (**b**) contour plot of the maximum SIF, tests, and mirrored tests plotted in the crack length space.

In order to validate the CCF, the authors tested eight aluminum specimens (Al-2023-T3 clad). There is a hole with a 2.5 mm radius in the middle of the coupon leading to two critical spots. Its width, length, and thickness is 35 mm, 90 mm and 1.8 mm respectively. The specimens were subjected to a pure tensile cyclic load with $F_{max}=16\;kN$. Figure 2a depicts the test setup. During the entire test, a camera was filming the coupon in order to measure the crack lengths on the left and right-hand side of the hole right before the final fracture. Figure 2b presents the field of the maximum SIF in the crack length space, which is computed by using the compounding method [35]. Moreover, the plot shows the critical crack lengths measured by the camera. The blue points represent the measured values. Since the test setup as well as the specimen are symmetric, the results could also have been illustrated inversely e.g., by looking from the backside at the specimen. The red points indicate this in Figure 2b.

Furthermore, Figure 2b shows that all points lie between an SIF of $1213\;MPa\sqrt{mm}$ and $1456\;MPa\sqrt{mm}$. Therefore, the test results confirm the assumption of the CCF, which is based on the fracture criterion with a maximum SIF equal to the fracture toughness. This is valid for cracks propagating towards free edges and not if the fracture criterion should disregard cracks, which rapidly propagate but end up in e.g., a hole.

## 4. Application to a Full-Scale Structure

**Figure 3:**
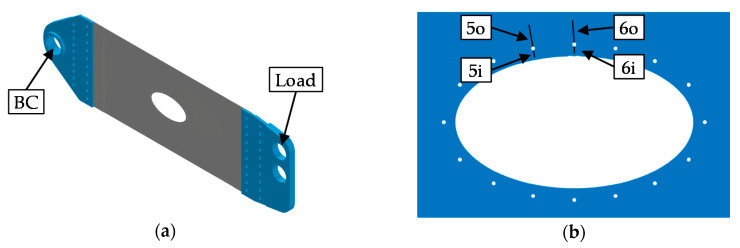
(**a**) CAD-Model of the demonstrator with its connections; (**b**) nomenclature of the critical spots and exemplary crack configuration.

In this, the method proposed in Section 2 is applied to a structure shown in Figure 3a. This demonstrator structure resembles the lower surface of an aircraft wing. The grey structure in Figure 3a represents the demonstrator. Its material is Al 2024-T351, which is a typical aluminum alloy used in such aircraft components. The demonstrator is 1.2 m long, 0.5 m wide, and 1.6 mm thick. It is subjected to a cyclic load spectrum, which resembles real flight scenarios consisting of soft to harsh flights (A, B, C and D flights). In order to perform this fatigue test, the blue steel connections in Figure 3a link the demonstrator with the fatigue test machine. In the fatigue test, a pin through the upper right hole fixes the assembly to the test machine such that no torque can be transmitted. The load is applied at the left hole of the assembly. The boundary conditions lead to a skewed load to resemble the torsion of a wing.

The corresponding finite element model consists of 123,203 nodes and 120,233 elements. Due to the dimensions of the demonstrator and the in-plane loading, the plane–stress condition is fulfilled, i.e., within good approximation there is no stress in the thickness direction. To achieve cheaper computations, linear, 2D elements are used. Most of the elements are Abaqus CPS4 elements with four nodes. The chosen element formulation for triangle elements is CPS3. The size of the elements reaches from 6mm in less stressed areas to0.06 mm in strongly stressed areas, at holes 5 and 6. These small elements were created in the context of a convergence analysis. In order to ensure that the boundary conditions correspond to the supports of the real test, the nodes around the holes marked with “BC” and “Load” are constrained (see Figure 3a). The pins, which connect the demonstrator to the experimental setup, are represented by rigid connections between the nodes on the surface of the holes and an additional node in the middle of each hole. The node in the middle of the hole labeled with “BC” is constrained in the horizontal and vertical direction, and the node in the middle of the hole labeled with “Load” is constrained perpendicular to the acting force direction.

The following subsections show the steps carried out to determine zones which are suitable to detect cracks by strain sensors.

### 4.1. Detection Zones of the Demonstrator

*Identify critical spots.* First of all, the fatigue critical spots must be determined in order to compute the detection zones. This is done by conducting an FEA and evaluating the stress ranges by the rainflow counting scheme according to the ASTM E 1049 Standard [36] (p. 287–293). After transferring the stress history to an alternating stress sequence according to the Haigh-Diagram [33] (p. 29) with the corresponding equations found in [37] (p. 98), Miner-Original [32] and the S-N-curve of Al 2024–T351 map the damage index to the corresponding location. As a result of this procedure, four positions are judged to be critical spots as these exhibit damage values, which are a magnitude greater than other positions. Figure 3b shows the four positions at holes 5 and 6, which are called 5i, 5o, 6i, and 6o (i: inner, o: outer). The origin of each crack is assumed to be in the middle of the finite element, which exhibits the maximum stress. The direction of the crack is chosen perpendicular to the direction of the principal stress. The fracture toughness $K_{C}$ is assumed to be $1044\;MPa\sqrt{mm}$ [38] (p. 413).

*Sample crack lengths.* In the next step, 2000 crack configurations ($a_{5i},a_{5o},a_{6i},a_{6o}$) are sampled randomly with a uniform probability distribution. The maximum value of the load spectrum is used as the applied load since, during service, it is always possible that the next load is the maximum load. The energy release rate G determined by FEA can be used to compute a corresponding SIF K, taking advantage of the plane–stress condition, which leads to Equation (5) [39] (p. 59). As a result, 2000 configurations with corresponding SIFs are known:

(5) $$K=\sqrt{G\;E}$$

*Train surrogate model.* Since there is no fast evaluation of SIFs for several interacting cracks, surrogate models, e.g., neural networks, are used to interpolate between sampled data. The inputs of each neural network are the four corresponding crack lengths, whereas the output of one neural network is the desired SIF corresponding to its crack location. The 2000 combinations of crack lengths and SIFs are used to train (75%) and test (25%) the surrogate model. All four neural networks have one layer with 24 fully-connected neurons. Table 1 summarizes the R^2^ value, the false positive rate (FPR), and the false negative rate (FNR) of all trained models, where false positive means that a crack is declared wrongly as critical. The result is considered to be sufficiently accurate.

*Sample critical crack configurations.* In the next step, the DCF is created by a Monte Carlo sampling. As explained in Section 2, the SIF serves as the fracture criterion. The critical value for the SIF, the fracture toughness, will be impinged with a safety factor to detect the crack before it gets critical and to absorb uncertainties. The fracture toughness $K_{c}=1044\;MPa\sqrt{mm}$ is reduced to $850\;MPa\sqrt{mm}$ according to Equation (1). Four crack lengths are created and serve as input for the four neural networks. If the maximum of the four computed SIFs is $\left(850\pm 8.5\right)\;MPa\;\sqrt{mm}$, this combination of four crack lengths $a_{c}$ is part of the DCF. As there are four cracks, the DCF is a hyperplane in the four-dimensional space of a.

**Figure 4:**
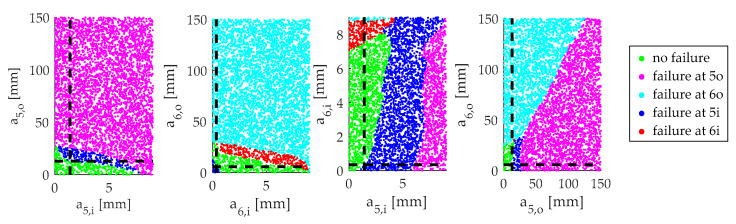
Sections of the space of a showing different failure regions.

Figure 4 shows four sections of the a-space, which visualize whether a combination of crack lengths is critical or not. The intersection of the dashed lines represents the point of interest in the a-space. The black dashed lines in one section diagram indicate the values of a used to generate the other section diagrams. Figure 4 depicts a point on the DCF since it is on the border to a state, where the crack 5i violates the SIF criterion. The second diagram from the left visualizes that the SIF of crack 5i becomes higher for reducing the length of crack 6i or 6o. This might be counterintuitive, but a longer crack 6i or 6o results in a smaller stress concentration at crack 5i by guiding the load path further from 5i away.

*Compute strain fields.* The next step is to determine the detection zones. To detect cracks with strain sensors, zones with a change of strain due to the cracks from the DCF have to be determined. For this purpose, the resulting strain fields have to be extracted from an FEA. In total, 200 strain fields were computed. The number was increased until the detection zones showed very limited changes. According to [40], an estimate based on 200 simulations has a confidence interval of at most ±7% at a 95% confidence level. The criteria for the crack detection zones are based on the absolute $\Delta \epsilon_{abs}$ and relative change of strain $\Delta \epsilon_{rel}$. The differences between the 200 strain fields and the crack free strain field are computed according to Equations (2) and (3).

*Identify detection zones.* To account for measurement errors and in order to identify the crack, the threshold on the relative change of strain is set to 10% and on the minimum absolute change of strain to 20 microstrains. The computation works as follows: The values for $\Delta \epsilon_{abs}$ and $\Delta \epsilon_{rel}$ are computed for every integration point of the FEA model. If a point fulfills both, conditions on $\Delta \epsilon_{abs}$ and on $\Delta \epsilon_{rel}$, the point is part of the detection zone for this specific crack configuration. After performing this procedure for all 200 configurations, one point is part of the detection zone, if it fulfills the conditions for all 200 configurations.

**Figure 5:**
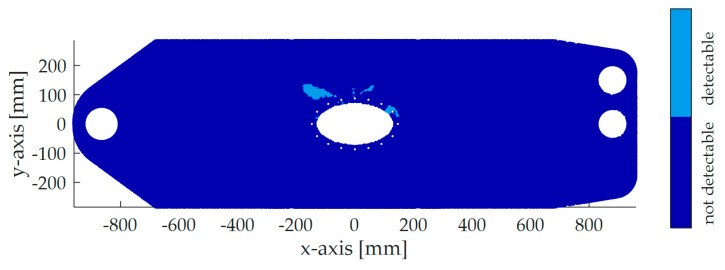
Detection map with crack detection zones for an application angle of 135°. All highlighted regions satisfy $\Delta \epsilon_{rel}\geq 0.1$ and $\Delta \epsilon_{abs}\geq 20$ microstrains.

Furthermore, the direction in which the values for $\Delta \epsilon_{abs}$ and $\Delta \epsilon_{rel}$ are computed, have a strong impact on the size of the determined detection zones. To increase these detection zones, the direction is optimized. Since strain sensors cannot be applied with arbitrary accuracy, in the optimization, the angle is varied discretely in 1-degree steps. Figure 5 shows the detection map for an optimized angle of 135° with respect to the *x*–*y* system.

### 4.2. Considering Crack Paths of the Demonstrator

As the crack can be detected not only at its critical state but also during its evolution, the detection zones can be extended by adding regions of smaller cracks.

**Figure 6:**
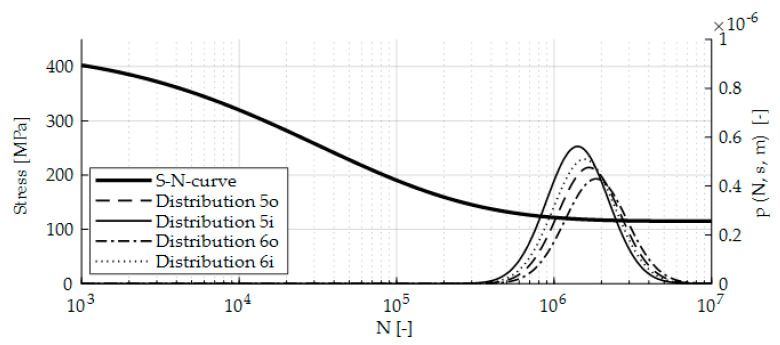
S-N-curve and crack initiation distribution.

*Sample initial cracks.* To compute crack paths, the number of cycles until crack initiation has to be identified. The proposed extension assumes the S-N-curve as the criterion for crack initiation. The thick, solid line in Figure 6 represents the used S-N-curve [41] for a 50% probability of failure. Since fatigue life is subjected to uncertainty, the number of cycles until crack initiation is modeled as a random variable. To deal with this uncertainty, the scatter is approximated by a probability density function (PDF):

(6) $$p\left(N,s,m\right)=\frac{\log_{10}\left(e\right)}{Ns\sqrt{2\pi }}exp\left[-\frac{1}{2}{\left(\frac{\log_{10}\left(N\right)-m}{s}\right)}^{2}\right]$$

Figure 6 shows the PDFs of the critical spots (5i, 5o, 6i, 6o) with their corresponding axis on the right. The authors used a log10 distribution with the scatter parameter s=0.197 [33] (p. 527) for all four critical spots and a log10 of the mean m, given by the S-N-curve (see Equation (6)). Finding the corresponding stress levels is similar to the approach for determining the critical spots. A safety factor of 3.0 [5] (p. 1149) is applied to the determined values of N to consider uncertainties in the computation.

**Figure 7:**
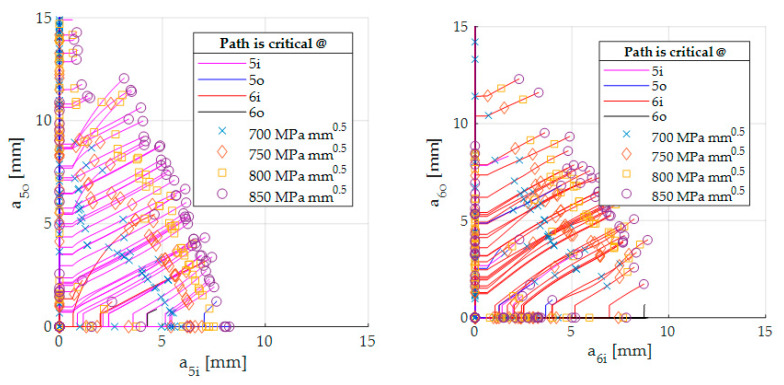
Crack paths with its discretization.

*Compute and discretize crack paths.* From these PDFs, the authors generated 200 configurations, each containing four (one for each critical spot) numbers of cycles until crack initiation. To apply the Paris law of Equation (4), an initial crack length has to be assumed. Since the Paris law is only valid for macroscopic cracks in classic fracture mechanics, the initial crack length must not be too small [42]. Here, the initial crack length is $a_{0}=0.635$ mm. This value is according to [43] (p. 30) the minimum detectable crack length if Eddy Current is used as the detection method. The material parameters of the Paris law are set to $m^{*}=2.92$ and $C=1.08\cdot 10^{-8}\;\frac{mm}{cycle\;{\left(MPa\;\sqrt{m}\right)}^{m^{*}}}$ [44] (p. 233). Furthermore, the load spectrum is simplified to a pure swelling load sequence according to the Haigh-Diagram. Now, for each generated array, a crack is initiated at the location for the smallest N. The crack growth is computed until the next bigger value of N of the realization array is reached. Then, the next crack is initiated with the initial crack length. This procedure is performed until one of the existing cracks reaches the critical SIF. It takes into account that several or only one cracks can lead to failure. Figure 7 shows the corresponding crack paths. The color of the lines represents which crack gets critical. Some crack paths lie on the *x*- or *y*-axis, indicating that no crack is initiated in one of these two displayed positions.

In the next step, the crack paths are discretized. Along every path, four crack lengths are determined by an SIF criterion. The points on the paths in Figure 7 indicate where the biggest value defines the DCF.

*Compute strain fields.* The crack lengths of the discretized paths are extracted to compute the strain fields. For each crack path, four strain fields are evaluated according to the discretization described above.

**Figure 8:**
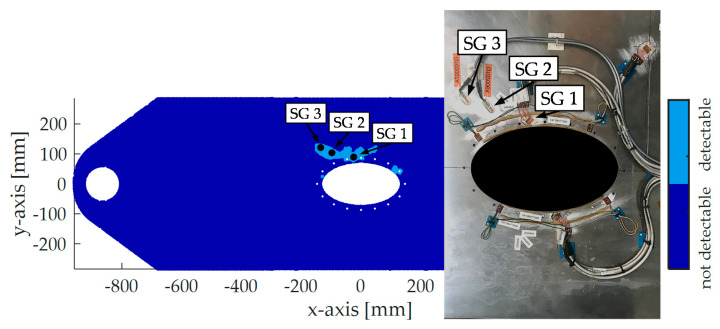
Detection zones in 130° resulted by extended method and applied SGs.

*Identify detection zones.* Since the crack can be detected on the way to its critical state, the union of the strain fields, which lie on the same crack path and fulfill the criteria on $\Delta \epsilon_{abs}=20$ microstrains and $\Delta \epsilon_{rel}=10\%$ describes the detection zones for the corresponding crack path. Finally, the intersection of all crack path detection zones determines the final detection map. Figure 8 shows the finally determined detection zones with respect to the mentioned criteria on $\Delta \epsilon_{abs}$ and $\Delta \epsilon_{rel}$. The angle, which maximizes the detection zones, is 130°. Like in Figure 5, a large detection zone is computed on the left side of the critical spots. In total, the detection zones in Figure 8 are bigger than the detection zones in Figure 5.

## 5. Measurement Results and Discussion

**Figure 9:**
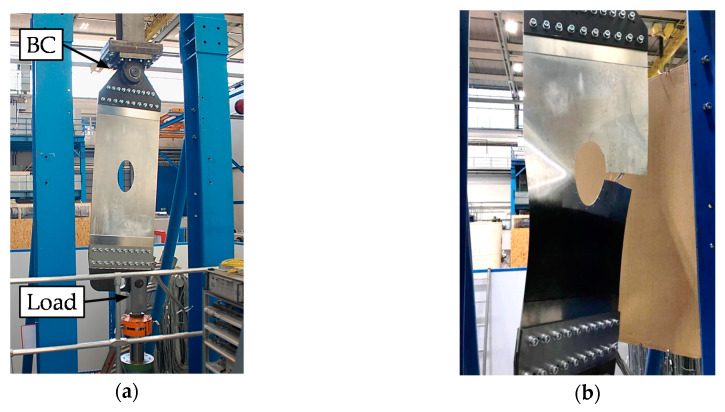
(**a**) test setup for demonstrator; (**b**) failed structure.

Figure 9a shows the test setup for the demonstrator. The structure is cyclically loaded at a slight angle to resemble tension and shear stress of a lower side of a wing. For the aim of this paper, three SGs are considered for SHM.

Figure 8 depicts the positions of the SGs:

- SG 1 is a strain gauge rosette and is used to record the strain in 0° (*x*-axis) and 130° direction. It is placed between the two critical holes of the structure, a position one would intuitively choose. In the 0° detection map, almost no detections zones are available. Only a region of about $1\;mm^{2}$ between the two critical holes is present, indicating that the detection with this position could be difficult. However, in a 130° direction, the SG shall detect the crack.
- SG 2 measures the strain in a 130° direction. It is quite close to the critical spots.
- SG 3 measures the strain in a 130° direction. Its location is further away from the critical spots than SG 2.

Figure 9b shows the failed structure. First, the demonstrator cracked through the inner part between hole 6 and the armhole. Then, the outer crack propagated and led to the final fracture. At about 8050 flights, the inner crack reached the fracture toughness and propagated rapidly through the inner part between hole 6 and the armhole. As it is required, that neither the inner nor the outer crack shall reach the fracture toughness, the SGs shall indicate the crack before 8050 flights with a 10% threshold.

**Figure 10:**
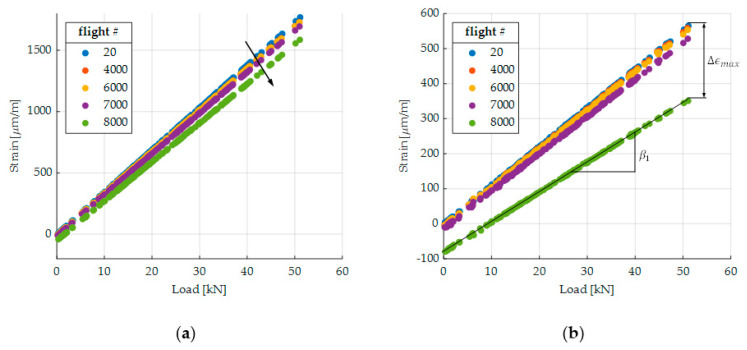

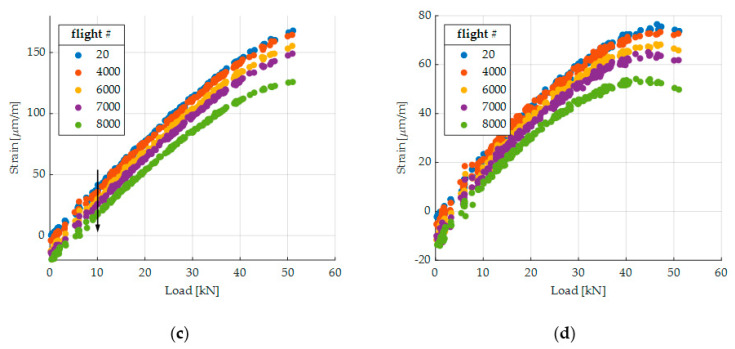
Strain evaluation of flight A at different times: (**a**) SG 1; (**b**) SG 1—130°; (**c**) SG 2; (**d**) SG 3.

In order to evaluate the evolution of the strain over time, we analyze the strain behavior of all A flights since this flight occurs most frequently. Figure 10 shows the measured strain of four different SGs over the applied load for different times. Due to fatigue-induced damage, the strain changes in two main ways: first, the load-strain slope, which is indicated in Figure 10a by the black array, and second, the strain level (see the black array in Figure 10c). Moreover, Figure 10 illustrates that SG 1 (Figure 10a) exhibits the smallest change of strain.

We consider two different measures for the evaluation over time: the load-strain slope $\beta_{1}$ and the maximum strain $\epsilon_{max}$ of an A flight. Both are indicated in Figure 10b. If the load is not known, e.g., in a real flight scenario, the load can be substituted by a remotely applied SG. The slope is determined for each A flight by computing the linear regression coefficient $\beta_{1}$ (Equation (7)), where $x_{i}$ and $y_{i}$ are the load levels and the corresponding strain levels, respectively. Furthermore, n is the total number of measured points during flight A and $\beta_{0}$ is the level at x=0:

(7) $$\left[\begin{array}{c} \beta_{0}\\ \beta_{1} \end{array}\right]={\left[\begin{array}{cc} 1 & x_{0}\\ \vdots & \vdots \\ 1 & x_{n} \end{array}\right]}^{-1}\left[\begin{array}{c} y_{0}\\ \vdots \\ y_{n} \end{array}\right].$$

**Figure 11:**
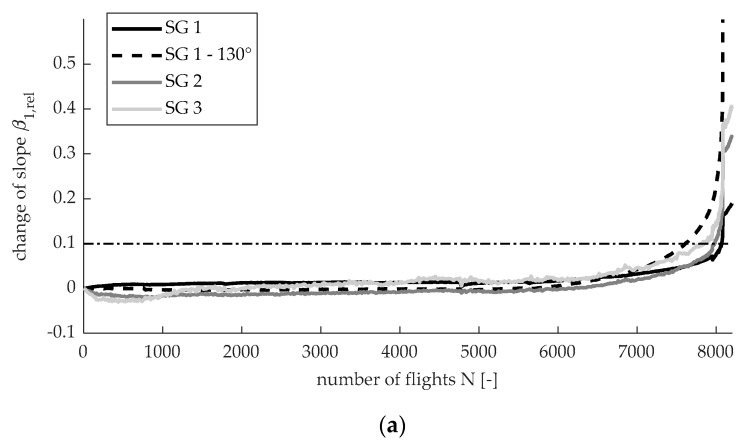

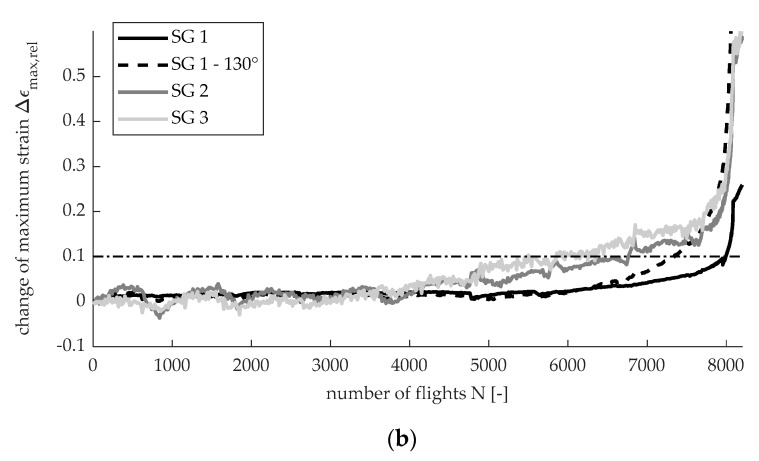
Evolution of change of strain: (**a**) change of slope; (**b**) change of maximum strain.

Figure 11 shows the measurements of SG 1, SG 1—130°, SG 2, and SG 3. The horizontal lines indicate the 10% threshold. Figure 11a displays the relative change of the slope $\Delta \beta_{1,rel}$ with respect to the slope of the first A flight. The relative change of the maximum strain $\Delta \epsilon_{max,rel}$ with respect to the maximum strain of the first A flight is illustrated by Figure 11b. In addition, $\beta_{0}$ can be evaluated over time. However, as this value is approximately zero for the first flight, the relative change reaches high values and the threshold of 10% is therefore no more useful.

By comparing Figure 11a,b, one can see that the line corresponding to $\Delta \epsilon_{max,rel}$ is wigglier. This might be due to the temperature dependency of the maximum strain, whereas the slope is independent of the temperature. Furthermore, based on the 10% threshold, the crack is detected earlier with respect to $\Delta \epsilon_{max,rel}$, since it accumulates both fatigue-induced effects, the change of the slope, and the shifting of the strain level. One negative aspect of $\Delta \epsilon_{max,rel}$ is that it could be affected by sensor drift. Moreover, in a real flight scenario, the maximum strain will change from flight to flight. However, the maximum strain can be substituted by a strain at a certain load or strain value (of a remotely applied SG), which is high and occurs frequently. Table 2 summarizes the time of detection for both measures. On average, the crack can be detected 880 flights earlier based on $\Delta \epsilon_{max,rel}$. However, one might apply a different threshold to the slope $\Delta \beta_{1,rel}$ since its line is smoother.

Corresponding to the 10% threshold for $\Delta \epsilon_{max,rel}$, SG 1—0°, SG 1—130°, SG 2, and SG 3 detect the crack at 7999, 7360, 6780, and 5860 flights, respectively. Therefore, SG 1—130°, SG 2, and SG 3 are able to detect the crack significantly earlier. This validates the method and shows that SGs, which are further away from the critical spots, can detect cracks. SG 3 detects the crack earlier than SG 2. This might be a result of the lower initial strain at SG 3, which leads to an earlier 10% change. Furthermore, it can be concluded that SG 1—0° is not a good sensor position since the detection zones are too small for applying an SG. The test confirms this, since SG 1—0° triggers only when the rapid crack propagation happens. Interestingly, SG 1—0°, which is the closest to the critical spots and thus might be the most tempting SG, leads to very poor monitoring, as it would stop the test only when rapid crack propagation begins. Therefore, putting strain sensors close to the defect is not always appropriate. Additionally, the SG 1—130° shows a 10% change of strain about 700 flights before rapid crack propagation, which validates the computed direction and the detection zones of Figure 8. Moreover, according to the proposed method, only one SG can detect four different cracks in this example. Therefore, the method can lead to a lower number of sensors and thus save application time and costs. However, since the demonstrator test is only one possible realization, there is no proof that one SG can detect all possibilities of cracks. Further tests must be carried out in order to generalize these results.

By comparing both proposed methods, i.e., with and without considering crack paths, Figure 5 and Figure 8 show that both methods generate large detection zones. Furthermore, the two angles are almost the same. In contrast to the first method, the second determines detection zones close to the critical spots. This is because crack configurations, which exhibit smaller SIFs, are also considered. In total, the method, which considers the crack path, leads to bigger detection zones. However, it is computationally more expensive, since strain fields for several points on the crack path have to be evaluated. Moreover, the crack paths have to be computed.

## 6. Conclusions

First, the authors confirm that the CCF can be used to accurately predict failure. Several coupon tests show that the final fracture for a cyclically loaded structure, where the cracks propagate towards a free edge, happens on a CCF, where at least one SIF is equal to fracture toughness.

Furthermore, the computed detection zones show that it is possible to detect the criticality of one among several cracks by only one sensor. Therefore, the method helps to identify the smallest number of necessary sensors in order to detect cracks. Since the first method depicts most of the detection zones, the authors recommend for simplicity the less expensive method. The demonstrator test confirms the computed detection zones by three SGs, which indicate cracks before rapid crack propagation occurs. Consequently, the results show that strain sensors should not only be positioned close to expected defects, which would be the intuitive thing to do. The proposed method generates a reliable dataset and supports a trustworthy SHM system.

Finally, two different strain measures are discussed. In this study, we could detect the crack earlier based on the maximum strain during a flight than on the load-strain slope.

## Figures and Tables

**Table 1:** Evaluation of neural networks with test set.

Target	R^2^	FPR	FNR
SIF 5i	0.968	1.21%	1.01%
SIF 5o	0.953	1.38%	0.92%
SIF 6i	0.973	1.02%	0.82%
SIF 6o	0.954	1.72%	1.72%

**Table 2:** Flight # of detection for two different measures.

Measure	SG 1	SG 1—130°	SG 2	SG 3
$\Delta \beta_{1,rel}$	8076	7620 *	7987	7836
$\Delta \epsilon_{max,rel}$	7999	7360 *	6780 *	5860 *

* until flight # 7800 only every 10th flight is evaluated.

## References

[1] Gobbato M., Kosmatka J.B., Conte J.P. (2014). A recursive Bayesian approach for fatigue damage prognosis: An experimental validation at the reliability component level. Mech. Syst. Signal Process..

[2] Farrar C.R., Worden K. (2006). An introduction to structural health monitoring. Philos. Trans. R. Soc. A: Math. Phys. Eng. Sci..

[3] Sikorska J., Hodkiewicz M., Ma L. (2011). Prognostic modelling options for remaining useful life estimation by industry. Mech. Syst. Signal Process..

[4] Rossow C.-C., Wolf K., Horst P. (2014). Handbuch der Luftfahrzeugtechnik.

[5] Henning F., Moeller E. (2011). Handbuch Leichtbau: Methoden, Werkstoffe, Fertigung.

[6] Grosse C.U., Ohtsu M. (2008). Acoustic Emission Testing.

[7] Lindley T., Palmer I., Richards C. (1978). Acoustic emission monitoring of fatigue crack growth. Mater. Sci. Eng..

[8] Roberts T., Talebzadeh M. (2003). Acoustic emission monitoring of fatigue crack propagation. J. Constr. Steel Res..

[9] Omondi B., Aggelis D.G., Sol H., Sitters C. (2016). Improved crack monitoring in structural concrete by combined acoustic emission and digital image correlation techniques. Struct. Heal. Monit..

[10] Grondel S., Delebarre C., Assaad J., Dupuis J.-P., Reithler L. (2002). Fatigue crack monitoring of riveted aluminium strap joints by Lamb wave analysis and acoustic emission measurement techniques. NDT E Int..

[11] Verstrynge E., De Wilder K., Drougkas A., Voet E., Van Balen K., Wevers M. (2018). Crack monitoring in historical masonry with distributed strain and acoustic emission sensing techniques. Constr. Build. Mater..

[12] McAlorum J., Fusiek G., Rubert T., Niewczas P. Concrete fatigue experiment for sensor prototyping and validation of industrial SHM trials. Proceedings of the Concrete fatigue experiment for sensor prototyping and validation of industrial SHM trials.

[13] Silva-Muñoz R.A., Lopez-Anido R. (2009). Structural health monitoring of marine composite structural joints using embedded fiber Bragg grating strain sensors. Compos. Struct..

[14] Imai M., Nakano R., Kono T., Ichinomiya T., Miura S., Mure M. (2010). Crack Detection Application for Fiber Reinforced Concrete Using BOCDA-Based Optical Fiber Strain Sensor. J. Struct. Eng..

[15] Yao Y., Tung S.-T.E., Glisic B. (2014). Crack detection and characterization techniques-An overview. Struct. Control. Heal. Monit..

[16] Jinachandran S., Basu A., Li H., Xi J., Prusty B.G., Rajan G. (2019). The Study of the Directional Sensitivity of Fiber Bragg Gratings for Acoustic Emission Measurements. IEEE Sens J..

[17] Gangopadhyay T.K., Majumder M., Chakraborty A.K., Dikshit A.K., Bhattacharya D.K. (2009). Fibre Bragg grating strain sensor and study of its packaging material for use in critical analysis on steel structure. Sens Actuators A: Phys..

[18] Majumder M., Gangopadhyay T.K., Chakraborty A.K., Dasgupta K., Bhattacharya D. (2008). Fibre Bragg gratings in structural health monitoring—Present status and applications. Sens Actuators A: Phys..

[19] Zhang N., Davis C., Chiu W.K., Boilard T., Bernier M. (2019). Fatigue Performance of Type I Fibre Bragg Grating Strain Sensors. Sensors.

[20] Ciminello M., Boffa N.D., Concilio A., Memmolo V., Monaco E., Ricci F. (2019). Stringer debonding edge detection employing fiber optics by combined distributed strain profile and wave scattering approaches for non-model based SHM. Compos. Struct..

[21] Deraemaeker A., Preumont A., Kullaa J. Modeling and removal of environmental effects for vibration based SHM using spatial filtering and factor analysis. Proceedings of the IMAC XXIV.

[22] Fritzen C.P. (2005). Vibration-Based Structural Health Monitoring – Concepts and Applications. Key Eng. Mater..

[23] Sohn H., Farrar C.R. (2001). Damage diagnosis using time series analysis of vibration signals. Smart Mater. Struct..

[24] Preumont A. (1997). Vibration Control of Active Structures.

[25] Katsikeros C., Labeas G. (2009). Development and validation of a strain-based Structural Health Monitoring system. Mech. Syst. Signal Process..

[26] Sbarufatti C., Manes A., Giglio M. (2013). Application of sensor technologies for local and distributed structural health monitoring. Struct. Control. Heal. Monit..

[27] Munns T.E., Kent R.M., Bartolini A., Gause C.B., Borinski J.W., Dietz J., Elster J.L., Boyd C., Vicari L., Ray A. (2002). Health Monitoring for Airframe Structural Characterization: NASA Technical Reports Server (NTRS). https://ntrs.nasa.gov/archive/nasa/casi.ntrs.nasa.gov/20020030899.pdf.

[28] Flynn E., Todd M.D. (2010). A Bayesian approach to optimal sensor placement for structural health monitoring with application to active sensing. Mech. Syst. Signal Process..

[29] Li B., Wang D., Wang F., Ni Y.Q. High Quality Sensor Placement for SHM Systems: Refocusing on Application Demands. Proceedings of the INFOCOM - IEEE Conference on Computer Communications workshops.

[30] Lecompte D., Vantomme J., Sol H. (2006). Crack Detection in a Concrete Beam using Two Different Camera Techniques. Struct. Heal. Monit..

[31] Clormann U.H., Seeger T. (1986). Rainflow-HCM. Ein Zählverfahren für Betriebsfestigkeitsnachweise auf werkstoffmechanischer Grundlage. STAHLBAU DER.

[32] Miner M.A. (1945). Cumulative Damage in Fatigue. J. Appl. Mech..

[33] Haibach E. (2006). Betriebsfestigkeit.

[34] Paris P., Erdogan F. (1963). A critical analysis of crack propagation laws. J. Basic Eng..

[35] Kuo A., Yasgur D., Levy M. (1986). Assessment of Damage Tolerance Requirements and Analyses-Task I Report.

[36] E ASTM (1985). Standard practices for cycle counting in fatigue analysis. Am. Soc. Test. Mater. Stand..

[37] Rennert R., Maschinenbau F.F. (2012). Rechnerischer Festigkeitsnachweis für Maschinenbauteile aus Stahl, Eisenguss-und Aluminiumwerkstoffen.

[38] Bucci R. (1979). Selecting aluminum alloys to resist failure by fracture mechanisms. Eng. Fract. Mech..

[39] Anderson T.L. (2005). Fracture Mechanics: Fundamentals and Applications.

[40] Lehar M., Zimmermann M. (2012). An inexpensive estimate of failure probability for high-dimensional systems with uncertainty. Struct. Saf..

[41] Handbuch L. (1978). Handbuch, L. Handbuch Struktur Berechnung (HSB). Industrie-Ausschufl Struktur Berechnungsunterlagen, Ausgabe C.

[42] Pugno N., Ciavarella M., Cornetti P., Carpinteri A. (2006). A generalized Paris’ law for fatigue crack growth. J. Mech. Phys. Solids.

[43] Ryschkewitsch M.G. (2008). Nondestructive Evaluation Requirements for Fracture Critical Metallic Components.

[44] Bergner F. (2000). A new approach to the correlation between the coefficient and the exponent in the power law equation of fatigue crack growth. Int. J. Fatigue.

